# Performance and Morphology of Waterborne Polyurethane Asphalt in the Vicinity of Phase Inversion

**DOI:** 10.3390/ma17133368

**Published:** 2024-07-08

**Authors:** Chengwei Wu, Haocheng Yang, Xinpeng Cui, Yachun Chen, Zhonghua Xi, Jun Cai, Junsheng Zhang, Hongfeng Xie

**Affiliations:** 1 MOE Key Laboratory of High Performance Polymer Materials and Technology, School of Chemistry and Chemical Engineering, Nanjing University, Nanjing 210023, China; 522022240039@smail.nju.edu.cn (C.W.); 522023240043@smail.nju.edu.cn (H.Y.); 15299908527@163.com (X.C.); 2 Sobute New Materials Co., Ltd., Nanjing 211103, China; 13585153616@163.com; 3 Experimental Chemistry Teaching Center, School of Chemistry and Chemical Engineering, Nanjing University, Nanjing 210023, China; xizh@nju.edu.cn; 4 Public Instrument Center, School of Chemistry and Chemical Engineering, Nanjing University, Nanjing 210023, China; caijun@nju.edu.cn

**Keywords:** waterborne polyurethane, asphalt emulsion, phase separation, phase inversion, mechanical properties

## Abstract

Waterborne polyurethane asphalt emulsion (WPUA) is an environmentally friendly bituminous material, whose performance is highly dependent on the phase structure of the continuous phase. In this paper, WPUAs in the vicinity of phase inversion were prepared using waterborne polyurethane (WPU) and asphalt emulsion. The chemical structures, thermal stability, dynamic mechanical properties, phase-separated morphology and mechanical performance of WPUAs were studied. Fourier-transform infrared (FTIR) spectra revealed that there are no –NCO bonds in either the pure WPU or WPUAs. Moreover, the preparation of WPUA is a physical process. The addition of WPU weakens the thermal stability of asphalt emulsion. WPU improves the storage modulus of asphalt emulsion at lower and higher temperatures. The glass transition temperatures of the WPUA films are higher than that of the pure WPU film. When the WPU concentration increases from 30 wt% to 40 wt%, phase inversion occurs; that is, the continuous phase shifts from asphalt to WPU. The WPUA films have lower tensile strength and toughness than the pure WPU film. However, the elongations at break of the WPUA films are higher than that of the pure WPU film. Both the tensile strength and toughness of the WPUA films increase with the WPU concentration. Due to the occurrence of phase inversion, the elongation at break, tensile strength and toughness of the WPUA film containing 30 wt% WPU are increased by 29%, 250% and 369%, respectively, compared to the film with 40 wt% WPU.

## 1. Introduction

With the global increase in road infrastructures and the harsh environmental impact, more and more attention has been paid to the development of environmentally friendly and sustainable road materials. A good example of these materials is asphalt emulsion (AE), which was first used in the early 20th century. Nowadays, 5–10% of paving-grade asphalt is used to produce asphalt emulsion [1].

Asphalt emulsion is an oil-in-water (O/W) emulsion, in which small asphalt droplets disperse in the continuous water phase with the aid of the anionic, cationic or non-ionic emulsifier. As the basic ingredient, asphalt binder makes up 50–75% of an asphalt emulsion [2]. The existence of low-viscosity water extremely lowers the viscosity of asphalt. In this case, the viscosity of asphalt emulsion is in the range of 0.05–1 Pa⸱s at 60 °C, which is considerably lower than asphalt itself (10–400 Pa·s). This viscosity allows asphalt emulsion to be applied in cold paving technologies, such as binders for cold recycling mixtures, bond coats, chip seals and microsurfacings [3,4]. Compared to hot-mix asphalt, cold paving techniques are less hazardous and more economical and environmentally friendly due to their fewer emissions, energy consumption and oxidation of asphalt [5,6]. When used for on-site or in-place techniques with no energy usage and emissions related to the transportation and drying of aggregates, asphalt emulsion gains particularly positive benefits.

However, the performance of asphalt emulsion used in cold technologies is not high enough for use under heavy traffic. One way to solve this problem is through the modification of asphalt emulsion with polymers [7,8]. Polymer modification improves the performance of asphalt emulsions, such as adhesion and the resistance to rutting, thermal and fatigue cracking. Polymer-modified asphalt emulsion has lower life cycle costs since the durability of unmodified asphalt emulsion is enhanced and pavement distress is mitigated [9,10].

There are three routes to produce polymer-modified asphalt emulsion: latex-modified emulsions by co-emulsification, also known as latex pre-addition, or post-addition, and emulsions of polymer-modified asphalt. Some polymer emulsions or latexes, including neoprene, natural rubber (NR) and styrene-butadiene rubber (SBR), which are available during the production of rubbers, have been used to modify asphalt emulsion [9,11].

Apart from neoprene, NR and SBR latex, many other polymers, including thermosetting polymers, are available as waterborne with growing environmental awareness [12]. As one of the fastest growing polymers, polyurethane (PU) finds ubiquitous applications in virtually all areas, such as foams, adhesives, elastomers, coatings and sealants [13,14,15]. Because of the health and environmental concerns regarding volatile organic compounds (VOCs) raised by solvent-borne polyurethane (SPU), waterborne polyurethane (WPU), also known as polyurethane dispersion (PUD), has attracted significant interest since the early 1990s [16]. WPU containing polyurethane nanoparticles dispersed in water exhibits similar or superior performance to solvent-borne polyurethane. Furthermore, WPU has numerous advantages over SPU, such as appreciable molecular weight, controlled viscosity, stiffness, user-friendly applicability and environmental friendliness [17,18,19,20].

Unlike thermoplastic neoprene, NR and SBR latex, waterborne polyurethane can be crosslinked, which endows the material with better adhesion and durability [21,22]. Recently, waterborne polyurethane has gained much attention for the modification of asphalt [23]. Zhao et al. [24] used WPU to modify asphalt binder. WPU improves the storage stability, workability and deformation resistance at high temperatures. However, the addition of WPU slightly weakens the fatigue resistance of the asphalt binder. Sheng et al. [25] prepared waterborne polyurethane asphalt (WPUA) by emulsifying PU-modified asphalt in soapy water. WPUA exhibits better ductility than styrene-butadiene-styrene (SBS)-modified asphalt. WPUA forms a stable multiphase structure through physical modification. Wei et al. [26] prepared recyclable WPU (RWPU) through dual dynamic disulfide and hydrogen bonds. Then, self-healing polyurethane asphalt emulsion was produced by mixing RWPU and asphalt emulsion. Strong physical crosslinking networks form in WPUA with the existence of WPU. WPUA shows good stress–strain healing efficiency (>73%). Besides neat asphalt emulsion, WPU has also been used in the modification of other polymer-modified asphalt emulsions. WPU (3–12 wt%) was utilized to modify SBR-emulsified asphalt. The addition of WPU enhances the softening point, creep recovery rate and rutting factor of the asphalt emulsion [27]. To improve the toughness of epoxy resin, WPU was introduced to waterborne epoxy asphalt emulsion. Xu et al. [28] reported that WPU improves the glass transition temperature (T_g_), toughness, elongation at break and low-temperature stress relaxation of the waterborne epoxy asphalt emulsion. It is well acknowledged that polymer-modified asphalts with a continuous polymer phase exhibit superior performance compared to those with a continuous asphalt phase [29,30,31]. The WPU concentration in all the above research is no more than 15 wt%, which makes asphalt the continuous phase with a weaker performance compared to WPU. Phase inversion takes place when the WPU concentration increases to a certain extent, which means that the continuous asphalt phase converts to the continuous WPU phase with higher performance.

The objective of this paper is to investigate the performance and morphology of waterborne polyurethane asphalt emulsion in the vicinity of phase inversion. To achieve this goal, WPUAs with WPU concentrations in the vicinity of phase inversion were prepared. Th performance and morphology of WPUAs were characterized using the following techniques. Firstly, the chemical structures of WPUAs were evaluated using Fourier-transform infrared (FTIR) spectra. Secondly, thermogravimetric analysis (TGA) was used to compare the thermal stability of AE, WPU and WPUA films. Thirdly, the dynamic modulus and T_g_ of the WPUA films were determined by means of dynamic mechanical analysis (DMA). Fourthly, the phase-separated microstructures of WPUAs were observed using laser scanning confocal microscopy (LSCM). Finally, the mechanical properties of the WPUA films in the vicinity of phase inversion were compared with those of the pure WPU film using a universal test machine.

## 2. Materials and Methods

### 2.1. Materials

Table 1 summarizes the detailed information on materials used in this paper. Table 2 lists the overview of CO-436. DMBA and PTMEG were dried at 130 °C in a vacuum before use. The water used was deionized (DI) water. All other solvents were used without further purification. Asphalt binder was provided by China Offshore Bitumen (Taizhou) Co., Ltd. (Taizhou, China). The properties of asphalt binder are summarized in Table 3.

### 2.2. Synthesis of WPU

**Figure 1:**
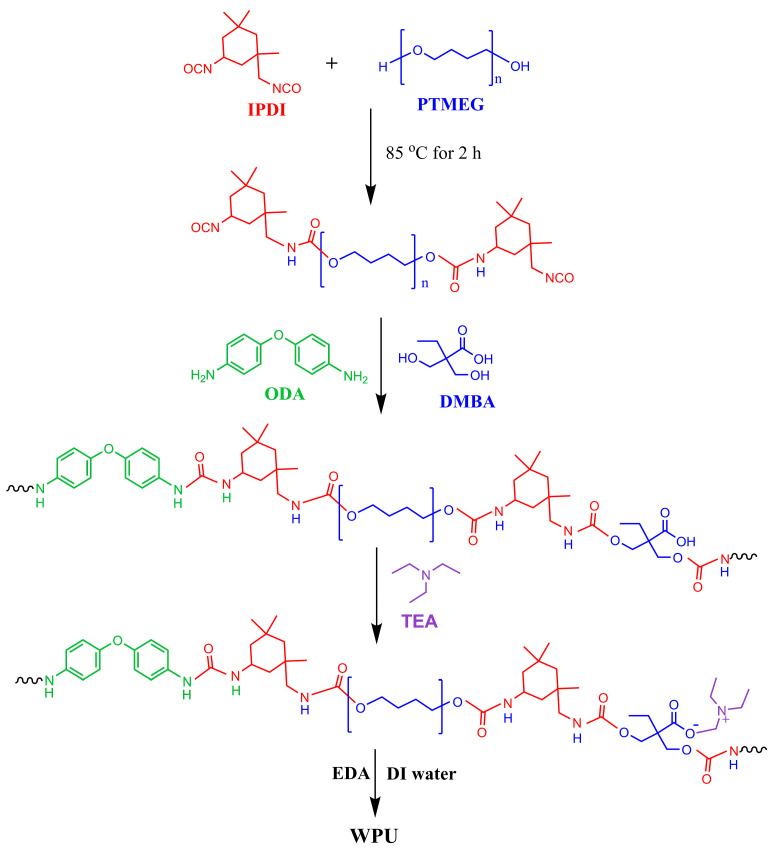
Synthesis procedure of WPU.

A total of 40 g PTMEG and 12.3 g IPDI were reacted in a three-necked flask at 85 °C for 1.5 h. After being cooled to 60 °C, 3 g DMBA dissolved in 10 mL acetone was introduced into the mixture and reacted for 1 h. Then, 1.94 g ODA dissolved in 10 mL was added and reacted for another 1 h. Finally, 2.04 g TEA, 0.2 g EDA and 73 g deionized water were added and stirred at 1000 rpm for 20 min. WPU with 45% solid content was procured after removing acetone in a vacuum. Figure 1 shows the synthesis procedure of WPU.

### 2.3. Preparation of Asphalt Emulsion

The asphalt binder was preheated at 150 °C for 2 h. A 5% emulsifier was introduced into DI water in a beaker. Then, the mixture was heated to 80 °C and agitated at 200 rpm for 30 min to prepare soapy water. Finally, the hot asphalt binder was slowly added to the soapy water with simultaneous agitation at 2000 rpm for 10 min. The asphalt–water weight ratio in the asphalt emulsion was 6:4.

### 2.4. Preparation of Waterborne Polyurethane Asphalt

**Figure 2:**
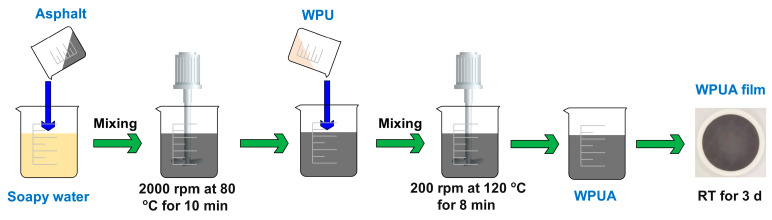
Schematic presentation of WPUA film preparation.

To prepare WPUA, both WPU and asphalt emulsion were heated to 120 °C and mixed at 200 rpm for 8 min. Afterward, the mixture was poured into a circular Teflon mold with a height of 5 mm and a diameter of 100 mm and kept at room temperature (RT) for 3 d to prepare the WPUA film. The asphalt contents in WPUAs were 0, 30, 40 and 50 wt%, respectively. Figure 2 presents the preparation procedure for the WPUA film.

### 2.5. Methods

#### 2.5.1. FTIR Spectroscopy

As one of the starting materials of polyurethane, isocyanate is toxic [37]. Furthermore, isocyanate can react with the functional groups in bitumen. Thus, FTIR spectra were used to evaluate the chemical structures, especially isocyanate groups, and the interaction between AE and WPU. FTIR spectra were obtained from an FTIR spectrometer (Alpha II, Bruker, Billerica, MA, USA) equipped with an attenuated total reflection (ATR). All spectra within the range of 4000–400 cm^−1^ were the average of 32 scans with a resolution of 4 cm^−1^.

#### 2.5.2. Confocal Microscopy

Phase separation usually occurs in polymer-modified asphalts. In addition, the performance of polymer-modified asphalts closely depends on the continuous polymer or asphalt phase [31]. Confocal microscopy is a powerful tool for observing the phase morphology of polymer-modified asphalts [38]. The phase-separated microstructures of waterborne polyurethane asphalt were studied on a Zeiss LSM 710 LSCM (Jena, Germany) with an Ar+ laser light of 488 nm. A minute drop of polyurethane asphalt emulsion was dripped onto a glass slide. Cover slides were placed on glass slides after removing the water on a hot stage. For each sample, three images with ×100 magnification were used to determine the average diameters and areas of dispersed phases.

#### 2.5.3. TGA

When being used as bond coats, polymer-modified asphalt adhesives generally undergo thermal shock from the upper hot bituminous mixtures [39]. In this case, the thermal stability of bond coats should be high enough to withstand the shock. In this paper, the thermal stability of the WPUA films was studied by means of TGA, which was performed on a TGA/DSC 1 instrument (Mettler-Toledo, Schwarzenbach, Switzerland). About 10 mg sample in an aluminum oxide crucible was tested at a ramping rate of 20 °C/min from 50 °C to 600 °C under the protection of N_2_ with a flow rate of 20 mL/min.

#### 2.5.4. Dynamic Mechanical Analysis

DMA provides information about the dynamic mechanical properties of polymeric materials, such as stiffness and the ability to dissipate energy as well as major glass transitions [40]. In this paper, DMA was used to evaluate the dynamic modulus and T_g_ of WPMAs. A dynamic mechanical analyzer (DMA + 450, 01 dB-Metravib, Limonest, France) was utilized to investigate the dynamic mechanical properties of the WPUA films. A WPUA film with dimensions of 2.5 mm × 1.5 mm × 2 mm was tested from −50 °C to 100 °C at 1 Hz and at a ramping rate of 3 °C/min.

#### 2.5.5. Mechanical Test

The final properties of WPUA depend on the flexibility and toughness of its film. An efficient selection of a WPUA formulation requires a basic understanding of its mechanical behavior and the factors that affect it [41]. The mechanical properties of WPU and WPUA films were determined on a universal testing machine (Instron 3366, Instron, Norwood, MA, USA) as per ASTM D638-22 [42]. The dog bone-shaped sample was tested at a rate of 200 mm/min at RT. Mechanical properties were obtained from the average value of five samples.

## 3. Results and Discussion

### 3.1. Evaluation of Chemical Structures

**Figure 3:**
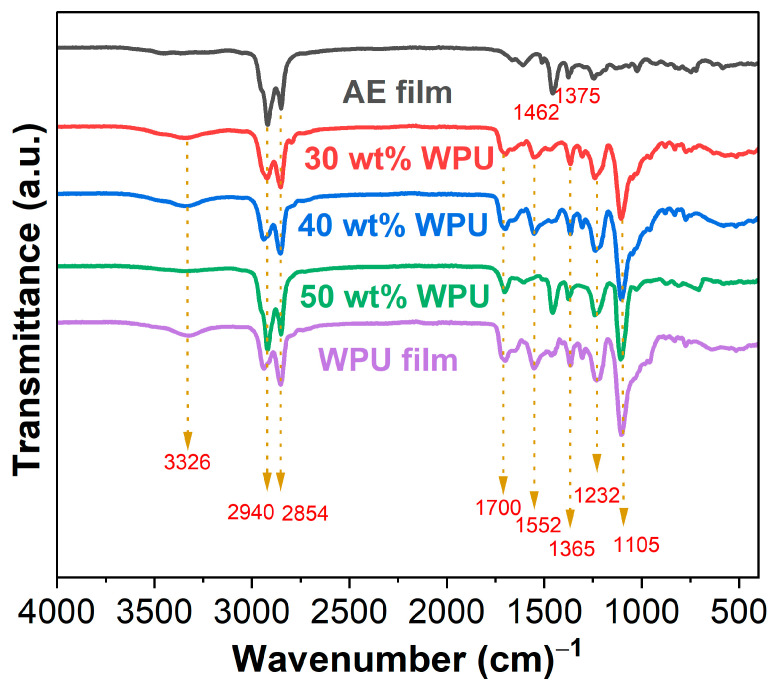
FTIR spectra of asphalt emulsion, WPU and WPUA films.

The FTIR spectra of asphalt emulsion, WPU and WPUA films are depicted in Figure 3. For asphalt emulsion, the double peaks at 2940 and 2854 cm^−1^ represent methyl (–CH_3_) and methylene (–CH_2_–) groups. –C=O of carbonyl, ester, aldehydes, ketones or amides is observed at 1671 cm^−1^. C=C of aromatics is shown at 1607 cm^−1^. The bonds at 1462 and 1375 cm^−1^ are attributed to aliphatic CH_2_ and CH_3_ [43]. For WPU, apart from the –CH_3_ and –CH_2_– groups at 2940 and 2854 cm^−1^, the stretching vibration of –NH is seen at 3326 cm^−1^. The peaks at 1700 cm^−1^ and 1552 cm^−1^ are related to –C=O and –CONH, respectively. Furthermore, the bonds at 1365 and 1232 cm^−1^ represent bending vibrations of methyl groups, while the stretching vibration of –COC– is shown at 1105 cm^−1^ [24,44]. In terms of WPUAs, all characteristic peaks of both asphalt emulsion and WPU exist. It is important to note that the peak at approximately 2250 cm^−1^ is not found in the spectra of either WPU or WPUAs, indicating that the toxic –NCO groups fully reacted during the preparation of WPU. Therefore, both WPU and WPUAs are environmentally friendly. Furthermore, it is known that the hydroxyl groups in asphalts react with –NCO groups [45]. Therefore, using non-solvent polyurethane to modify asphalt binder is a chemical process. However, the preparation of WPUAs is a complete procedure of physical mixing since all –NCO groups in WPU had been reacted, as shown in Figure 3. This indicates that there is no chemical interaction between WPU and asphalt emulsion.

### 3.2. Thermal Stability

**Figure 4:**
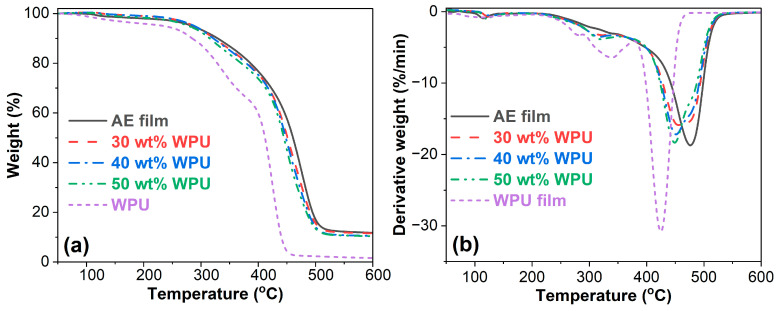
TGA (**a**) and DTG (**b**) curves of asphalt emulsion, WPU and WPUA films.

The TGA and DTG (derivative thermogravimetry) curves of asphalt emulsion, WPU and WPUA films are illustrated in Figure 4. The TGA curve of the WPU film is nearly beneath the AE film as shown in Figure 4a, indicating that the WPU film has poorer thermal stability than the AE film. As shown in Figure 4b, the AE film undergoes two-stage thermal composition, while the WPU film thermally degrades under three stages. For the AE film, the first stage takes place from 100 to 150 °C, assigned to the oxidation of the solvent within the film. The second decomposition stage in the temperature range of 250–550 °C is related to the degradation of larger molecules of asphalt binder [46,47]. In terms of the pure WPU film, the first decomposition stage takes place at 240–290 °C, relating to the pyrolysis of rigid segments of urethane and urea groups. The second decomposition stage occurs at 290–380 °C, assigned to the pyrolysis of soft segments. The third decomposition stage occurs at 380–480 °C, correlating to the gasification of residual organic compounds [48]. Because of the combined influence of AE and WPU, WPUAs also show a three-stage degradation, as shown in Figure 4b. More importantly, as shown in Figure 4a, the TGA curve of the WPU film is beneath those of all WPUA films over the entire range of temperatures, indicating that the WPU film has the worst thermal stability in comparison to both AE and WPUA films.

Table 4 summarizes the TGA and DTG results of WPU, asphalt emulsion and WPUA films. WPU has little influence on the onset decomposition temperature (T_onset_, the temperature at 95%) of the asphalt emulsion film when adding 30 wt% and 40 wt% WPU. However, with the addition of 50 wt% WPU, the T_onset_ of asphalt emulsion film declines due to the lower T_onset_ of the WPU film. The addition of WPU increases the maximum loss rate at the first stage (T^1^_max_) of the asphalt emulsion film. The WPU concentration has a negligible effect on the T^1^_max_ of the WPUA films. Nevertheless, the T^2^_max_ of the WPUA film with 30 wt% WPU is somewhat higher than those of the WPUA films with higher WPU concentrations. The T^3^_max_ of the WPUA film decreases with WPU concentration. The char at 600 °C of the WPU film is much lower than that of the asphalt emulsion film. In this case, the presence of WPU lowers the char at 600 °C of the asphalt emulsion film. In addition, the char at 600 °C of the WPUA films decreases with WPU concentration. The thermal stability of polymer-modified asphalts depends on the constitution and the interaction between polymer and asphalt binder [31]. The FTIR spectra reveal that there is no interaction between WPU and AE. Therefore, the thermal stability of WPUA is only dependent on its constitution. As shown in Figure 4a, the thermal stability of the neat WPU film is much poorer than that of the AE film. Thus, the inclusion of WPU sacrifices the thermal stability of the asphalt emulsion film. Moreover, the thermal stability of the WPUA films decreases with the increase in WPU concentration.

### 3.3. Dynamic Mechanical Properties

#### 3.3.1. Storage Modulus (E′)

**Figure 5:**
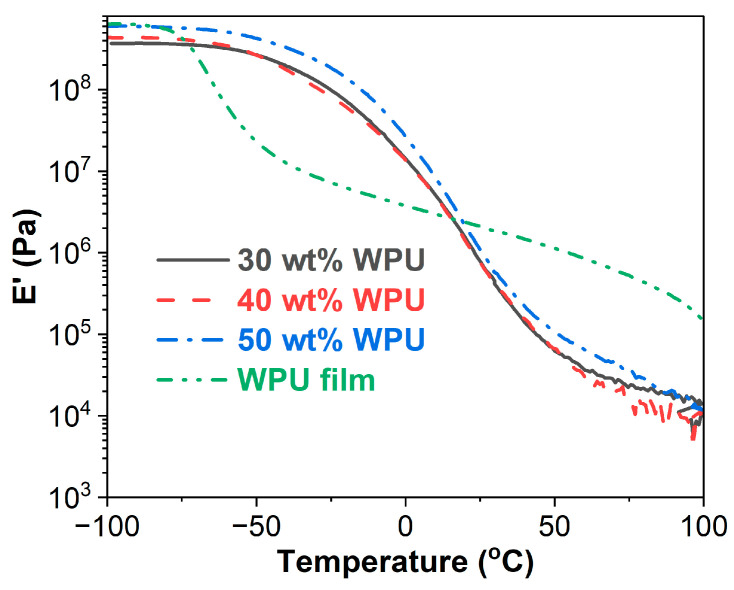
Storage modulus as a function of temperature for WPU and WPUA films.

The E′ versus temperature curves of the WPU and WPUA films are shown in Figure 5. The E′ of the WPU film is higher than those of the WPUA films in the temperature ranges of −100–−80 °C and 15–100 °C. However, in the temperature range of −80–15 °C, the E′ of the WPU film is lower than that of the WPUA films. In a DMA measurement, storage modulus relates to Young’s modulus of materials and indicates the elasticity and stiffness of materials [49]. This means that the WPU film has better elasticity and higher stiffness than the WPUA films in the temperature ranges of −100–−80 °C and 15–100 °C. However, an opposite trend is observed in the temperature range of −80–15 °C. For the WPUA films, the film with 50 wt% WPU has a higher E′ value than those with 30 wt% and 40 wt% WPU, while the films with lower WPU concentrations exhibit similar E′ values in the whole temperature range.

#### 3.3.2. Loss Modulus (E″)

**Figure 6:**
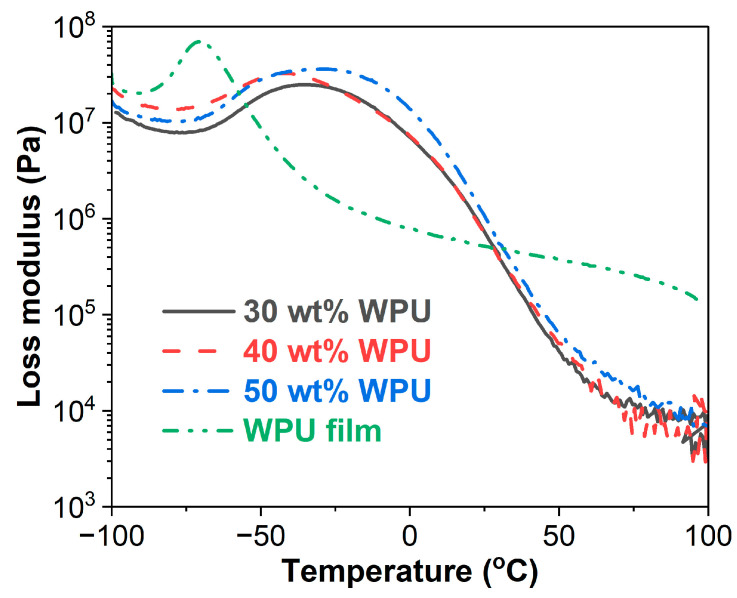
Loss modulus as a function of temperature for WPU and WPUA films.

Figure 6 presents E″ versus temperature curves of WPU and WPUA films. Similar to E′, the WPU film has higher E″ values than the WPUA films in the temperature ranges of −100–−60 °C and 30–100 °C, while the WPUA films have higher E″ values than the WPU film when the temperature is over 30 °C. In terms of the WPUA films, the film with 50 wt% WPU has higher E″ than the film containing 30 wt% WPU over the entire temperature range. The WPUA film with 40 wt% WPU has a higher E″ values than the one with 50 wt% WPU in the temperature range of −100–−50 °C. However, after about −30 °C, the film with 40 wt% WPU shows nearly the same E″ values as the one with 30 wt% WPU.

For polymeric materials, E″ represents the ability to dissipate energies and is sensitive to molecular motions, glass transitions, relaxation, morphology and structural heterogeneity [50,51]. In this context, the temperature of E″ is often chosen to represent the glass transition temperature [50]. As shown in Figure 6, the WPU film has a lower T_g_ than the WPUA films. For the WPUA films, there is no linear relationship with WPU concentrations. The T_g_s of WPUAs with 30 wt%, 40 wt% and 50 wt% WPU are −35.3 °C, −42.2 °C and −28.7 °C, respectively.

### 3.4. Morphology

**Figure 7:**
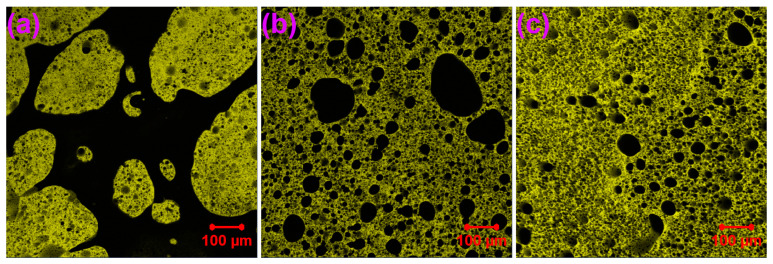
Confocal microscopy photographs of WPUAs with different WPU concentrations: 30 wt% (**a**), 40 wt% (**b**) and 50 wt% (**c**).

Figure 7 shows LSCM images of WPUAs. As shown in Figure 7a, for WPUA with 30 wt% WPU, some yellow WPU particles disperse in the black asphalt phase. That is to say, WPU particles act as the discontinuous phase, while asphalt behaves as the continuous phase. The dispersion of WPU particles is random. The size of some WPU particles is extremely large, indicating that the dispersion of WPU particles is near the co-continuous stage. With the further increase in WPU concentration, phase inversion occurs, meaning that WPU becomes the continuous phase, whereas asphalt particles turn to the discontinuous phase, as shown in Figure 7b,c.

To determine the average diameters and areas of asphalt phases, an image analysis software, Image-Pro Plus 6.5, was used [52]. Number-average and weight-average diameters (*D_n_* and *D_w_*) were calculated using the following equations [53]:

(1) $$D_{n}=\frac{\sum n_{i}D_{i}}{\sum n_{i}}$$

(2) $$D_{w}=\frac{\sum n_{i}D_{i}^{2}}{\sum n_{i}D_{i}}$$

where *n_i_* is the number of particles with the diameter of *D_i_*. Table 5 summarizes the average diameters, *PDI* (polydispersity index, *D_w_*/*D_n_*) and areas of asphalt phases. For WPUAs with a continuous WPU phase, the *D_n_*, *D_w_*, *PDI* and areas of asphalt phases decrease with WPU concentration. *PDI* indicates the uniformity of the discontinuous particles. The lower the *PDI* value, the more uniform the dispersed particles [54]. Thus, with the increase in WPU concentration, asphalt particles disperse more uniformly in WPUAs.

### 3.5. Mechanical Performance

**Figure 8:**
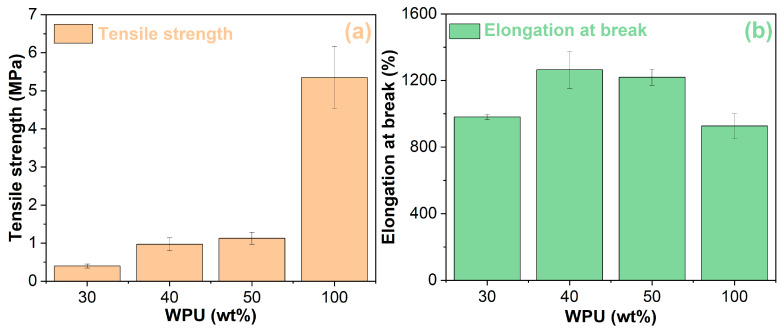
Mechanical properties of the WPU and WPUA films: tensile strength (**a**) and elongation at break (**b**).

The mechanical performance of the WPU and WPUA films is depicted in Figure 8. As shown in Figure 8a, the pure WPU film has a higher tensile strength than the WPUA films. In other words, from the perspective of the pure WPU film, the addition of asphalt emulsion greatly sacrifices its tensile strength. In the case of the WPUA films, the tensile strength increases with the increase in WPU concentration. Notably, there is a sharp increase in the tensile strength for the WPUA films when the WPU concentration increases from 30 wt% to 40 wt% because of the occurrence of phase inversion, as shown in Figure 7. A similar phenomenon also occurs in epoxy asphalts [55]. As shown in Figure 8b, both WPU and WPUAs have a high elongation at break, with a value over 900%. Furthermore, all WPUA films have higher elongation at break values than the pure WPU film. That indicates that the elongation at break of the pure WPU film is enhanced with the addition of asphalt emulsion. The WPUA film containing 40 wt% WPU has the maximum elongation at break, which is 29% and 4% greater than those of the WPUA films containing 30 and 50 wt% WPU, respectively. Due to the phase inversion, the elongation at break increases from 981% to 1265% when the concentration of WPU increases from 30 wt% to 40 wt%.

**Figure 9:**
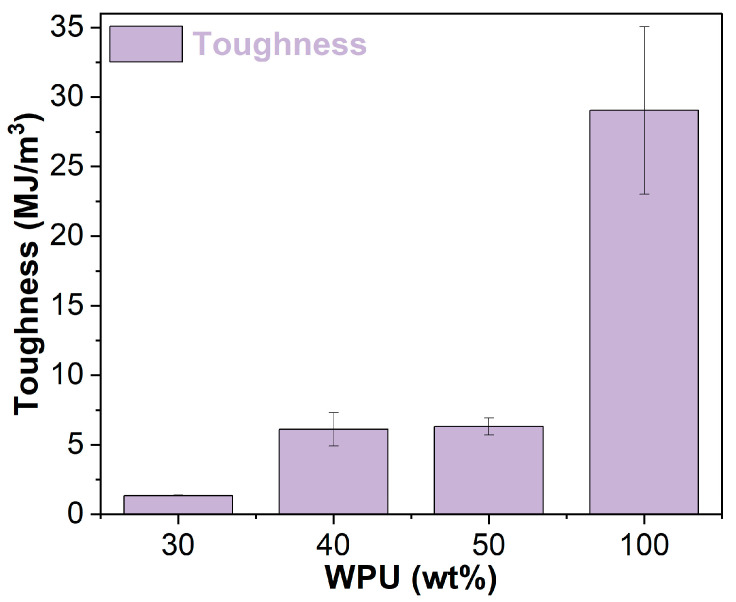
Toughness of the WPU and WPUA films.

Figure 9 shows the toughness of the WPU and WPUA films. The inclusion of asphalt emulsion significantly reduces the toughness of the pure WPU film. The toughness of the WPUA films decreases with lower WPU concentration. Due to the occurrence of phase inversion, the toughness of the WPUA film with 40 wt% WPU is 369% higher than that of the film with 30 wt% WPU.

## 4. Conclusions

Waterborne polyurethane asphalt emulsions with higher waterborne polyurethane concentrations were prepared and characterized. FTIR spectra revealed that the production of WPUA is a physical mixing procedure. Compared to the WPU film, the WPUA films have superior thermal stability. The thermal stability of the WPUA films decreases with the increase in WPU concentration. The WPU film has a lower storage modulus than the WPUA films between −80 °C and 15 °C. However, out of this temperature range, the WPU film shows higher storage moduli. In addition, the glass transition temperature of the WPU film is much lower than those of the WPUA films. Phase inversion in WPUAs takes place when the WPU concentration increases from 30 wt% to 40 wt%, resulting in the WPU conversion of the discontinuous phase to the continuous phase. From the perspective of the WPU film, the addition of asphalt emulsion greatly lowers the tensile strength and toughness of the WPU film. However, the incorporation of asphalt emulsion improves the elongation at break of the WPU film. In terms of the WPUA films, both tensile strength and toughness increase with WPU concentration. The occurrence of phase inversion has an important impact on the mechanical performance of the WPUA films.

In a word, environmentally friendly waterborne polyurethane asphalt with a continuous WPU phase has superior mechanical properties and good potential applications in bituminous pavements. Future research on the road performance of WPUA for pavement applications, such as binders for cold recycling mixtures, bond coats, chip seals and microsurfacings, needs to be conducted. The high PUA concentration has undoubtedly increased the cost of WPUA. However, high performance will result in the long-term durability of WPUA materials.

## Figures and Tables

**Table 1:** Detailed information on materials used in this paper.

Material	Abbreviation	Property	Manufacture
Polytetramethylene glycol	PTMEG	Molecular weight: 2000	Sichuan Tianhua Chemical Co. Ltd. (Luzhou, China)
Isophorone diisocyanate	IPDI	Industrial grade	Yantai Wanhua Polyurethanes Co., Ltd. (Yantai, China)
4,4′-oxydianiline	ODA	AR grade	Aladdin Company (Shanghai, China)
Bis(hydroxymethyl)butyric acid	DMBA	Purity: 99%	Sinopharm Chemical Reagent Co., Ltd. (Shanghai, China)
Triethylamine	TEA	Purity: 99%	
Ethylenediamine	EDA	Purity: 99%	
Ethoxy alkyl phenolic ammonium sulfate	CO-436		Jiangsu Haian Petroleum Chemical Factory (Haian, China)

**Table 2:** Overview of CO-436.

Property	Value
Reactive compound (%)	58–60
Color (Hazen)	<500
Relative density	1.065
Odor	Alcohol
Water (%)	20–25
Nonvolatile component (%)	60–64
Alcohol (%)	12–16
pH	6.5–7.5

**Table 3:** Properties of asphalt binder.

Property	Standard	Value
Penetration (25 °C, 0.1 mm)	ASTM D5-06 [32]	91.0
Ductility (10 °C, cm)	ASTM D113-07 [33]	93.0
Softening point (°C)	ASTM D36-06 [34]	46.3
Viscosity (120 °C, Pa·s)	ASTM D4402-06 [35]	787
Saturates (%)	ASTM D4124-09 [36]	16.7
Aromatics (%)		33.9
Resins (%)		44.7
Asphaltenes (%)		4.7

**Table 4:** TGA and DTG results of WPU, asphalt emulsion and WPUA films.

WPU (wt%)	T_onset_ (°C)	T^1^_max_ (°C)	T^2^_max_ (°C)	T^3^_max_ (°C)	Char at 600 °C (%)
0	285	116	476	-	11.8
30	288	124	323	456	11.5
40	288	123	318	451	10.5
50	278	121	318	449	10.4
100	234	281	340	425	1.6

**Table 5:** Average diameters, PDIs and areas of asphalt phase in WPUAs.

WPU (wt%)	*D_n_* (μm)	*D_w_* (μm)	*PDI*	Area (%)
30	-	-	-	55.8 ± 7.7
40	10.4 ± 0.6	19.5 ± 0.8	1.88	28.5 ± 2.9
50	8.8 ± 1.1	14.4 ± 1.0	1.64	23.3 ± 1.8

## Data Availability

All data are available in the manuscript.
